# Incorporating Disease and Population Structure into Models of SIR Disease in Contact Networks

**DOI:** 10.1371/journal.pone.0069162

**Published:** 2013-08-19

**Authors:** Joel C. Miller, Erik M. Volz

**Affiliations:** 1 Departments of Mathematics and Biology, Penn State University, University Park, Pennsylvania, United States of America; 2 Department of Epidemiology, University of Michigan, Ann Arbor, Michigan, United States of America; Centre de Physique Théorique, France

## Abstract

We consider the recently introduced edge-based compartmental models (EBCM) for the spread of susceptible-infected-recovered (SIR) diseases in networks. These models differ from standard infectious disease models by focusing on the status of a random partner in the population, rather than a random individual. This change in focus leads to simple analytic models for the spread of SIR diseases in random networks with heterogeneous degree. In this paper we extend this approach to handle deviations of the disease or population from the simplistic assumptions of earlier work. We allow the population to have structure due to effects such as demographic features or multiple types of risk behavior. We allow the disease to have more complicated natural history. Although we introduce these modifications in the static network context, it is straightforward to incorporate them into dynamic network models. We also consider serosorting, which requires using dynamic network models. The basic methods we use to derive these generalizations are widely applicable, and so it is straightforward to introduce many other generalizations not considered here. Our goal is twofold: to provide a number of examples generalizing the EBCM method for various different population or disease structures and to provide insight into how to derive such a model under new sets of assumptions.

## Introduction

The social contact network of a population plays a significant role in controlling the spread of directly transmitted infectious diseases. The accuracy of our predictions about the course of an epidemic or the effectiveness of an intervention is thus tied closely to our ability to accurately model the impact of the contact network on disease transmission. Two of the features that are most notable in network observations are that partnerships may have significant duration and different individuals have different numbers of partners. Recent work [1]–[3] has provided a modeling approach referred to as “edge-based compartmental modeling” (EBCM) which incorporates both partnership duration and heterogeneities in numbers of partners for susceptible-infected-recovered (SIR) epidemics. These studies find surprisingly simple equations governing the macroscopic dynamics as the disease spreads through random contact networks. The EBCM models are expected to be exact in the large-population limit when the population satisfies appropriate assumptions, and have been proved exact in the special case of static Configuration Model populations [4].

Mechanistic infection-spread models which explicitly incorporate interactions between infected and susceptible individuals typically fall into one of two classes. We will refer to the most-common of these as “mass-action” models. In the simplest mass action model of a homogeneous population, the probability that two individuals 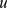 and 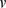 interact in a short time interval is the same for any pair of individuals, and does not depend on previous interactions. In more complex versions, we may stratify the population by demographic group (or other “type”). The probability that 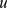 and 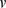 interact in a short time interval is given by the expected number of interactions between a pair of individuals in each of their groups but is independent of whether they have interacted previously. This allows us to capture biased mixing among children and adults or simply heterogeneities in numbers of partners *e.g.*, [5]–[9].

An important but often unrecognized assumption of mass-action models is that they implicitly assume partnerships have zero duration, so that at any moment the partners are randomly chosen from a large pool of potential partners. We can highlight the significance of this assumption by considering a married couple. These models assume that if a man gives his wife influenza, then an arbitrarily short time later he has a new wife who may still be susceptible and his original wife has a new husband who may be susceptible. The deterministic equations that result are exact for a population that satisfies these assumptions, but the failure of the assumptions may have important implications. In real populations, we expect that over time, infected individuals tend to infect those surrounding them, leading to local depletion of susceptibles. We cannot use these models to study any effect which depends on the duration of a partnership. For example, we cannot use mass-action models to investigate the effect of concurrency, *i.e.*, having partnerships that overlap in time, on the HIV epidemic because the mass-action equations assume that all partnerships are fleeting.

The majority of mechanistic models that are not mass action models are instead network-based. Rather than assuming partnerships have negligible duration, usually (but not always) these assume partnerships are permanent. Many of these use “pair approximations” [10]–[14]. Pair approximation models typically derive equations for the number of susceptible-susceptible and susceptible-infected partnerships, and often (but not always) assume that the population has homogeneous degree. The equations that arise are exact, but rely on knowledge about longer-range connections, such as the number of susceptible-susceptible-infected triples in the population. Typically an approximation is used whereby the frequency of each type of triples is expressed in terms of the pairs. For a commonly-used model network (the Configuration Model network, the simplest network model which allows for heterogeneous degree) it is possible to estimate the number of triples of various types exactly so long as the central individual is susceptible [14], [15] and the initial conditions are small. If the central individual is infected or recovered, the usual estimates for the number of triples are incorrect, but these do not feed back into the equations for the pairs and so the calculations for the pairs remain correct. These models frequently require many equations, with the number of equations being 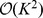 where 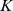 is the number of distinct degrees in the population. Although it has recently been shown that the number of equations can be reduced dramatically without introducing errors, the approach is not obvious [14], [15], and the more obvious simplifications introduce errors. With the reduction in dimension coming from [14], [15] the equations reduce to the equations of the EBCM approach.

Other exact mathematical models exist that also allow for epidemics in (Configuration Model) networks having heterogeneous degree, but typically the number of equations is proportional to the maximum degree of the network or the square of the maximum degree [16], [17]. If however we are willing to assume all individuals have the same number of partners, it is possible to write down a relatively simple system of equations that captures epidemic dynamics [18]. Other approaches allow us to capture the impact of dynamic partnerships, but these assume very uniform behavior across the population and occassionally assume no more than one partnership at a time [19]–[22].

The EBCM approach presented in [1] avoids many of the problems of other approaches, and is thus more flexible. We emphasize that this approach allows for heterogeneous degree in the underlying network. It directly leads to a simple system of equations which can be proven to exactly predict the disease dynamics in the large-population limit for a Configuration Model network [4]. The resulting system of equations has only a single governing differential equation. Subject to a few restrictions, we can adapt the assumptions so that the underlying population model differs from a Configuration Model network. The model can be adapted to include dynamic partnerships with only a small increase in complexity. Despite these advantages over existing approaches, the model retains some of the existing weaknesses. It makes very simple assumptions about the disease progression and additionally assumes that the only distinguishing feature of an individual is the number of partners. Thus it misses a large number of features: there may be a preferential direction to transmission (*e.g.*, a blood donor can infect, but not be infected by, the recipient); some individuals may be more or less susceptible to infection or more or less infectious per partnership than others (*e.g.*, some may withdraw from society when ill while others may continue as normal); there may be biased mixing by age or by behavior (school children preferentially contact one another over adults [23], or people with many sex-partners may preferentially select partners who also have many partners); there may be multiple modes of transmission (*e.g.*, IV needle sharing, heterosexual contacts, and homosexual contacts have different contact structures and different transmission probabilities [24]); the disease may have multiple phases of infection, each with different transmission rates (*e.g.*, the acute phase of HIV is widely believed to be more infectious than the longer-lasting chronic phase [25]); or even, the partnership dynamics may evolve in response to knowledge about infection status (*e.g.*, “serosorting” in response to HIV). These and many other features have been previously studied through mathematical models.

In this paper our goals are to show that many of the effects that have been studied by other approaches under the assumption that partnerships have zero duration can be captured by the EBCM approach to incorporate the effect of long-lived partnerships and to provide guidance to other researchers working with similar questions. The resulting equations are simpler than those found by other methods, do not require approximations inherent in many competing approaches, and allow us to account for heterogeneity in numbers of partners. The flexibility of this approach allows us to develop exact models for a wide range of different situations. Our scope is to show that for each effect considered the method results in a simple system of equations which accurately reproduces simulated epidemics rather than to perform a detailed investigation of the impact of each effect. We begin by demonstrating the model in the simplest static network with minimal structure. We next provide derivations for the cases described above, summarized in table 1, which by themselves form a “recipe book” for use by other modelers. The list is by no means exhaustive, and it is not difficult to combine different effects. More generally these may be used as exemplar models which can guide the derivation of models for other behaviors not considered here. We focus primarily on static networks and assume a very small initial condition; the generalization to dynamic networks is straightforward following [1], and the generalization to allow larger initial conditions is also straightforward following [26]. For our final example of serosorting we must turn to a dynamic network model as it is fundamental to the process. We finally give a discussion of limitations of the EBCM method, giving examples for which there appears to be no simple model. We make no claim that we are the first to study these effects, most of which have been studied in a mass-action setting already. Rather, this is the first simple approach allowing us to write down exact models for these and similar effects in a network setting.

**Table 1:** Models investigated in this article.

Model	Brief Description	Section
Directed Networks	Model for a disease in which some partnerships are not symmetric in terms of disease risk.	2.2.1
Heterogeneous Individuals	Model for populations with heterogeneities in infectiousness and/or susceptibility that do not correlate with population structure	2.2.2
Assortative mixing by type	Model for populations with demographic groups that have heterogeneities in infectiousness and/or susceptibility and partner selection is affected by an individual's group.	2.2.3
Multiple transmission modes	Model for a disease that can be transmitted by more than one type of behavior and the network structure induced by each behavior is different.	2.2.4
Multiple infectious stages	Model for a disease which has several infectious stages of possibly varying duration or infectiousness.	2.2.5
Serosorting	Model for dynamic network where edges break or are created at rates dependent on the status of partners.	2.3.1, 2.3.2

The edge-based compartmental models considered here. All of these except serosorting are presented using the (static) Configuration Model network structure. Serosorting is presented in two different dynamic network contexts.

## Method and Results

### 2.1 The basic model

The static network epidemic models we present here are all generalizations of the basic Configuration Model (CM) network epidemic model. To set the stage, we first define a CM network [27]–[30]. Because we will use the same underlying approach, we also briefly describe the underlying method of [1]. Generalizing the models we develop to dynamic networks is straightforward following [1].

**Figure 1:**
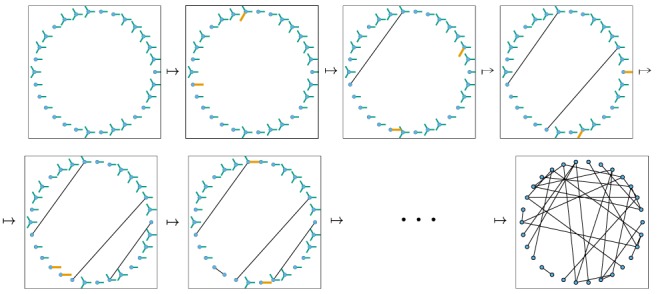
Sample generation of a Configuration Model network. The steps to generate a Configuration Model network with 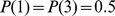. (Top left) Each node/individual is independently assigned either 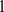 or 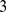 stubs with equal probability. (Successive plots) Pairs of stubs are randomly chosen and joined into edges until no stubs remain (bottom right). In the limit of a large population, about half of the individuals have 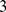 partners, about half have just 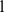 partner, and a random partner of a given individual is three times as likely to have 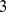 partners versus just 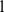. The number of triangles in the network is small; there is little clustering. For this 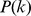 we have 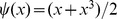.

In a CM network, each individual is represented by a *node* which is connected to other nodes by *edges* which can transmit disease. To construct the network, each node is assigned a number of edges (its *degree*) 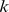 with probability 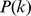. The edges connect randomly to one another using proportional mixing, so that the probability of selecting a partner of degree 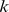 is 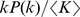 where 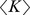 denotes the average of 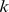. The generation of a CM network is illustrated in figure 1. It is convenient to define
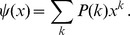
Note that 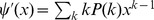 and 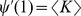.

We define 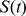 to be the proportion of the population still susceptible at time 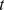, 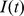 to be the proportion infected at time 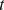, and 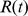 the proportion recovered at time 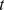. We make the assumption that these change deterministically at the population scale. By assuming that the population scale dynamics are deterministic, we are making one key assumption that we highlight here. When we consider a single individual 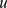 in multiple realizations of the epidemic, the time of infection of 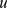 or even whether 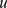 is ever infected is a random event. However, if the disease dynamics are deterministic at the population scale, then the details of when 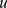 is infected and who 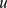 might later infect must not matter at the population scale. This is analgous to the “price-taker” assumption of economics in which, for example, a small farmer could not produce enough wheat to affect the price of wheat, and so the farmer must take whatever price the market is offering. The value of the price-taker assumption mathematically is that it allows us to ignore the impact an individual firm has on the broader market and just focus on the impact of the market on the firm's actions. In our case, the corresponding assumption allows us to focus our attention on the impact of the epidemic on 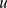 and expicitly ignore the impact of 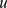 on the epidemic. Because 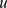 cannot affect the dynamics of the epidemic, we can modify 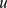 by not allowing it to transmit onwards without affecting the dynamics. When we prevent 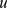 from transmitting onwards, it has no impact on the status of 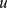, but it simplifies the calculation of the status of 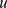 since we can then ignore correlations of partners of 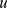 that are arise from transmission through 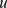.

Consider a random *test node* 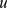 chosen uniformly from the population. We alter 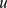 so that if infected, 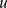 does not transmit to its partners. Because 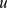 is chosen randomly, the probability that it is susceptible, infected, or recovered at a given time must match 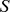, 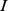, and 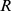. Thus our calculation proceeds by calculating the probability 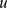 is in each state. We focus our attention on finding the probability 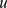 is susceptible. Once we know it, we are able to determine 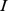 and 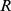 by 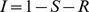, 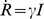.

We use 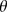 to be the probability that a random partner 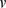 of 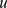 has not transmitted infection to 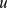. We break 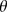 into three parts: 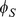, 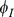, and 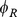, which are respectively the probability 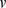 is still susceptible, the probability 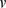 is infected but has not transmitted to 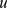, and the probability 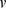 is recovered and did not transmit to 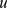. We have 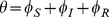.

**Figure 2:**
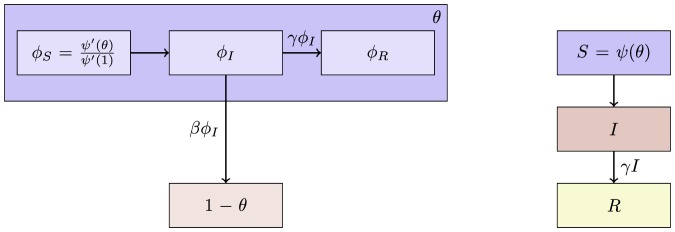
Flow diagram for Configuration Model networks. Given a test individual 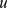 and a random partner 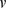, the probability 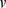 has not transmitted to 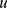 is 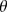. We divide 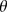 into three compartments based on the status of 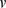: 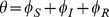 where the subscript gives the status of 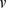. The flow diagram on the left gives the flux between these subcompartments within 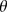 and the flux from 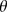 to 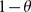 (which comes specifically from the 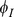 subcompartment of 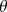). The flow diagram on the right shows the flux of individuals through the 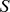, 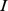, and 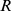 compartments. To find 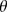, we must find 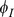, which is 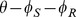. We find 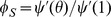. Because the flux into 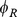 and 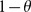 are proportional, we can find 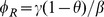. Thus 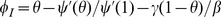, and we are able to find a differential equation for 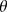 in terms of 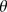. To find 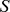, 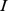, and 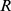, we note that the probability 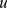 is in each state is equal to the proportion of the population in each state, so the susceptible proportion is equal to the probability that 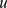 is susceptible, which is 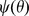. We determine 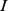 by 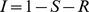 and 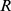 by 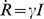.

The flow diagram of figure 2 gives the fluxes between the various compartments. Because the infection rate within a partnership is 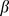 and the recovery rate of an individual is 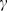 it is relatively straightforward to see that the fluxes from 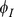 to 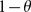 and 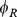 are 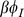 and 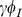 respectively. The calculation of the flux from 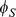 to 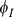 is less obvious. We have two options. We can calculate the flux directly or we can simply calculate 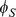 explicitly as was done in [1], in which case we can avoid the flux calculation altogether. This second option is simpler, and we use it here.

We have
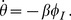
(1)The remainder of our derivation focuses on finding 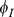. Because 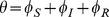, we have 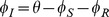. Thus we simply need to calculate 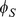 and 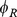 in terms of 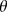 to find 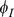 in terms of 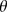. To calculate 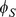, we use the fact that the probability a partner 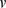 has degree 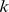 is 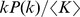. Since 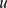 is prevented from infecting 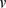, the probability 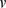 is susceptible given its 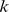 is 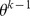. Thus 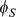 is a weighted average of this, 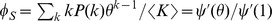.

To calculate 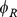, we look at figure 2. The fluxes into 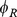 and into 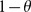 are proportional to one another, with the proportionality coefficient 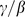. Since both variables begin at 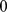, this means 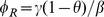. Consequently
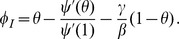
This leads to
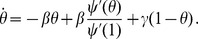
Thus we have the system of equations
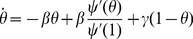
(2)

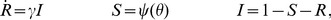
(3)which has just two ordinary differential equations (ODEs). In fact equation (2) does not depend on equation (3), so the system is governed by the single ODE (2).

We show the variables and their definitions in table 2.

**Table 2:** Variables and equations for the basic EBCM approach.

Variable	Definition	Equation
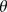	The probability that 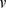 has not transmitted to 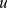 .	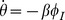
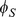	The probability that 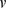 has not transmitted to 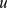 and is susceptible.	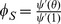
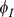	The probability that 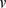 has not transmitted to 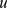 and is infected.	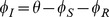
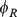	The probability that 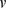 has not transmitted to 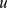 and has recovered.	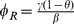
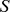	The probability 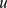 is susceptible or equivalently the proportion of the population that is susceptible.	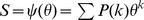
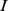	The probability 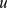 is infected or equivalently the proportion of the population that is infected.	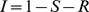
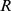	The probability 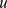 is recovered or equivalently the proportion of the population that is recovered.	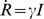
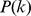	The proportion of individuals within the population having degree 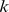 .	Not Applicable

Equations and variables assuming a negligibly small initial proportion infected. In each case 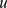 is a randomly chosen test individual (prevented from transmitting to others) and 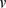 is a random partner of 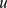.

#### 2.1.1 Initial Condition

Our derivation has implicitly assumed that the epidemic is started by an infinitesimally small initial condition, so that as 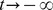, we find 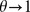, 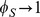, 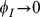, and 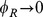. By focusing on linear terms, we find that the vector of 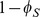, 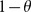, 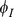, and 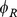 can be expressed in terms of an eigenvector of the associated matrix times 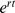 for some 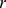. Thus if we know any one of these at 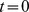, we can find the other variables by assuming that it has converged to this eigenvector. More generally, if the initial condition is not small, we cannot assume that it converges to the eigenvector before nonlinear terms become important. In this case, we need to account for the initial condition explicitly. This is discussed in [26]. The derivations below all assume small initial condition and that the epidemic is above the critical threshold, but the approach of [26] can account for a larger initial condition in each case or the behavior if the disease is not able to invade.

#### 2.1.2 Dynamic Networks

We can adapt the approach above to dynamic networks with relatively little difficulty. The main addition is that we have additional fluxes from and to the various 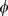 compartments as edges break or are formed. We must include some additional variables showing the proportion of all edges that belong to nodes of a given status as this determines the probability that a new edge is with a susceptible, infected or recovered node. Details are in [1], [31].

#### 2.1.3 Generalizing the model

In the remainder of this section, we generalize the model for many static network situations, and in section 2.3 we discuss some generalizations specific to dynamic networks. The basic approach is to consider a random test node 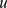 which is prevented from causing infection. Then consider the edges which could transmit infection to 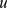. We determine the probability the edges have not transmitted to 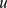, which may depend on 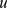, the partner, or details of the partnership. Our approach to determining this probability is the same as above. Once we know the probability any given edge has not transmitted to the test node, we can calculate the probability that the test node is susceptible, from which we can calculate the proportion of the population that is susceptible, infected, or recovered.

Our approach is fairly general and will apply to a wide range of populations. The generalizations we have here are by no means exhaustive, and many of the effects we consider here could be combined. The main property we require of the population is that the partners of a given individual are independent of one another. More precisely we require that we be able to assume that if we know the population scale details of the epidemic, then knowing the status of one partner of the test individual 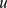 gives no information about the status of another partner of 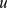.

### 2.2 Epidemics on generalized static networks

#### 2.2.1 Directed Networks

There are a number of realistic scenarios where infection can transmit in only one direction. Examples include blood transfusions, a food handler infecting a consumer, and even a patient infecting a doctor where they come into contact only because of the patient's infection. The probability and final size of epidemics for this scenario have been studied previously [32], but not the dynamics. Other researchers have investigated the dynamic spread of disease through asymmetric networks using pair-approximation based techniques [33]. The resulting pair-approximation model relies on a large number of equations. The model we derive here predicts the dynamics of an epidemic in a directed network, requires only a handful of equations, and is exact for the assumed network class.

We can investigate the dynamics in almost the same manner as before. Assume that the network consists of both directed and nondirected edges. The disease can transmit along a directed edge only following the edge direction, while nondirected edges may be followed in either direction. Let 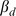 and 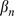 denote the rate of transmission along directed and nondirected edges respectively. Recovery occurs at rate 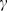 regardless of how infection was received.

**Figure 3:**
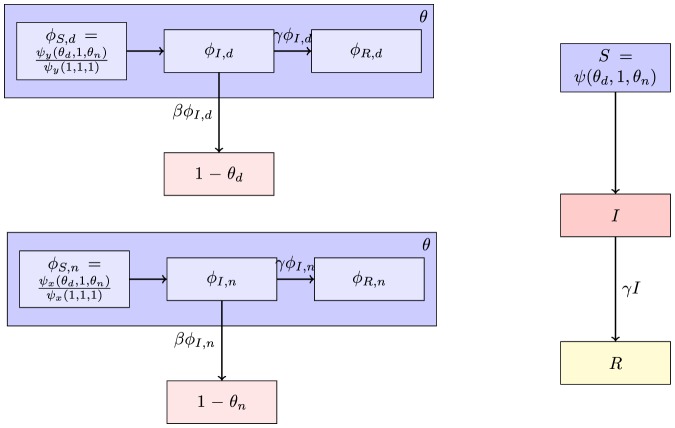
Directed CM model. Flow diagram for a network with directed and nondirected edges. We consider the two edge types separately. The evolution of edges is similar to figure 2. We can assign different infection rates for each edge type.

We refer to edges pointing to a node of interest as *in-directed*, and those pointing away as *out-directed* edges. The probability of having 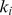 in-, 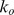 out-, and 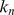 nondirected edges is given by 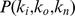. We define
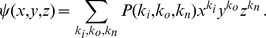
We again consider a random test node 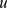 which is prevented from causing infection. We define 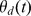 and 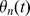 to be the probability an in-directed edge or nondirected edge to 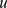 respectively has not transmitted infection to 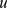. The probability that 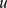 is still susceptible is 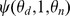. We use the variables 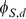, 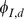, and 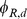 to be the equivalent of 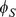, 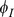, and 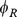 seen before for in-directed edges. Following the same approach as before we arrive at the flow diagrams in figure 3.

Consider a partner 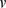 with a directed edge to 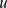. Because of how 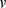 is chosen, the probability it has 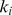 in-, 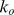 out-, and 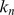 nondirected edges is 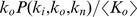. The probability that 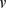 is still susceptible is 
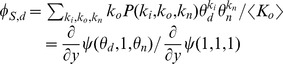
. The probability that 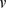 has recovered without infecting 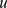 is 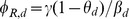. Because 
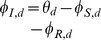
 we have 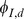 in terms of 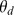 and 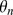. This gives us 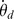 in terms of 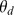 and 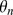. A similar expression holds for 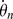. We have
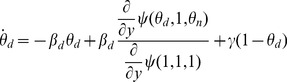
(4)

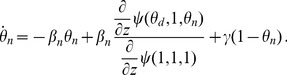
(5)To this we add
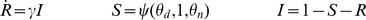
(6)to give us the proportion of susceptible, infected, and recovered individuals.

If the system only has directed edges, then we can drop 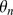 from the analysis and 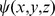 reduces to 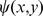. Such a model could be used to study the impact of superspreaders where the probability of receiving infection from an infected node is similar for all nodes (in-degrees are similar), but some nodes have many more partners to infect than others (high variance in out-degree). With minor modifications, we can adapt this method to edges which may transmit in both directions but have asymmetric transmission rates.

**Figure 4:**
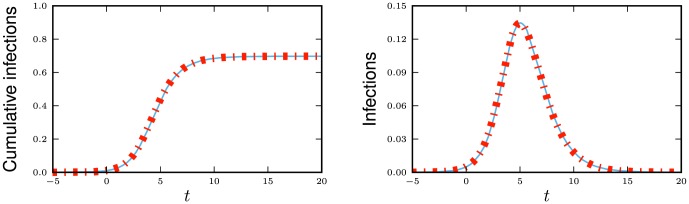
Directed CM example. Results for the directed networks described in section 2.2.1 using 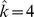, 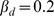, and 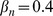. We choose 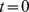 to correspond to 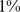 cumulative incidence. Theory (dashed) correctly predicts dynamics of simulations in a population of 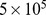 individuals (solid).

We now demonstrate the equations in a concrete example. Consider a population for which the average in-degree, out-degree, and nondirected degrees are each 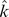 as follows: Each node has in-degree 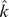. The out-degree 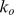 is uniformly chosen from 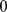 up to 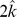 inclusive, and the nondirected degree is 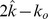. For this population,
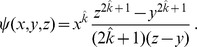

Figure 4 shows results for 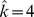, 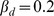, and 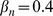.

#### 2.2.2 Heterogeneous infectiousness and susceptibility

Assume now that there is a parameter 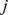 which measures a node's ability to become infected and cause infection, but does not influence the contact structure of the population. We refer to the value of 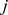 for a node as its *type*, and the probability a node has a given type 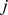 is 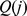. Although we assume the type is discretely distributed, it could be continuous with no significant complications. The recovery rate 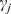 of a type-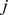 node and the transmission rate 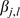 from a type-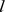 node to a type-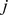 node are type dependent. The final size for a special case of this model where a node's infectiousness and susceptibility are uncorrelated is given in [34]. Similar problems have been considered for well-mixed mass action populations (*e.g.*, [35], [36] and many others).

Consider a random test node 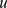 of type 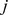. Let 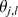 denote the probability that an edge from a type-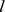 partner 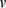 to 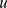 has not transmitted infection from 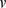 to 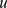, and similarly 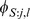 the probability 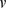 is still susceptible, 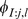 the probability 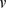 is infected but the edge has not transmitted, and 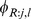 the probability that 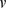 has recovered without transmitting. We define 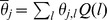 as the probability that a random edge to 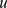 has not transmitted infection to 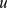. We use the original definition of 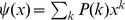.

**Figure 5:**
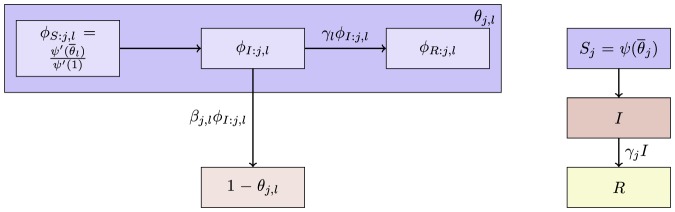
Heterogeneous infectiousness/susceptibility model. We separate nodes by type 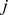, but assume that 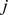 has no effect on connectivity. Both infectiousness and susceptibility may depend on 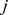. We must consider edges between each pair of types 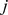 and 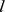 separately. The evolution of edges is similar to before.

We find that 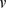 is susceptible with probability 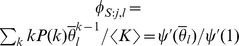. We also find that 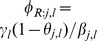. Then the flow diagram in figure 5 shows that
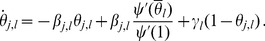
(7)The probabilities a type-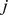 node is still susceptible, infected, or recovered satisfy
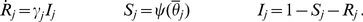
(8)The total population in each state is given by
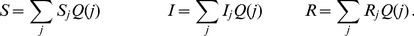
(9)


We now demonstrate the equations in a concrete example. One application of this model is to the impact of a partially effective vaccination. Vaccination generally reduces the susceptibility of a node, but could either increase or decrease the infectiousness of a node by reducing the severity of symptoms (less sick individuals may shed less virus but also maintain stronger contact intensity while symptomatic). If only part of the population is vaccinated, then the population can be divided into those who have or have not received vaccination.

**Figure 6:**
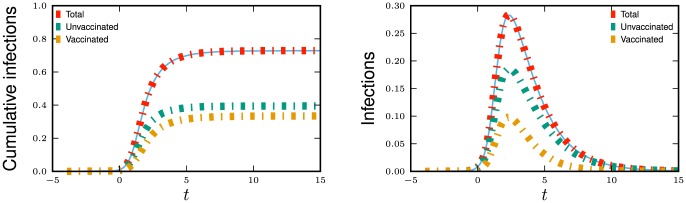
Heterogeneous infectiousness/susceptibility example. Epidemics spreading in a population for which half have received a leaky vaccine described in section 2.2.2. Vaccinated individuals are half as infectious and half as susceptible. We choose 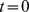 to correspond to 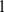
% cumulative incidence. Simulations in a population of 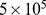 individuals (solid) and theory (dashed) are in good agreement.

Consider a population with a negative binomial degree distribution 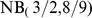 with size 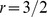 and probability 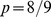, giving an average degree of 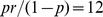 and variance of 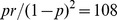. For a negative binomial distribution 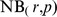 we have 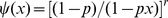, so
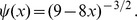
Assume that half of the population has received a leaky vaccine such that vaccinated nodes have reduced susceptibility, and — if infected — reduced infectiousness and infection duration. Let 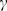 be the rate of recovery for unvaccinated nodes and 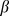 the rate of infection between unvaccinated nodes. Vaccinated nodes recover at rate 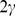, and the rate of infection between a vaccinated and unvaccinated node (in either direction) is 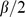 while the rate of infection between two vaccinated nodes is 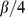. The vaccine is distributed uniformly. Results for 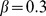, 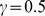 are shown in figure 6.

#### 2.2.3 Populations with assortative mixing by type

In many instances there is biased mixing between or within demographic groups, and the transmission/recovery parameters for the different groups may differ. For example, the spread of influenza is strongly affected by the increased level of mixing and increased infection rates between children. Many sexually transmitted diseases are strongly affected by differences in mixing rates and risk behavior among MSM and heterosexual groups. For this reason it is useful to have a model accommodating different levels of mixing within and between groups. Others have applied a pair-approximation approach for which assortative mixing is dependent on degree [10]. Here we generalize to also allow for assortative mixing that results from other demographic features such as age. The model we derive is equivalent (though simpler) to that of [24].

Assume that the population is made up of 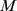 groups, and let 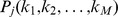 denote the probability a node of group 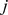 has 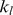 partnerships with nodes of group 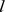 for 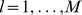. To simplify notation, we denote this by 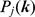 where 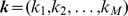. We similarly set 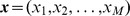 and use 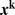 to denote 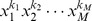. We set
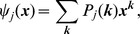
and let 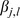 be the rate of transmission across an edge from group 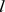 to group 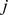. We similarly define 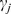 to be the recovery rate of a group 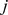 node.

We define 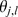 to be the probability an edge to a test node in group 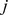 coming from a group 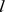 node has not transmitted infection. If our test node 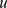 is of type 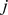, then the probability that a partner 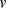 of type 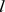 is still susceptible is 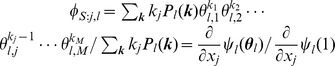 where 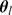 denotes the vector 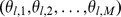 and 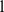 denotes the vector 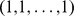. We can also show that 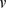 has recovered without transmitting to 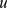 with probability 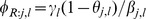.

**Figure 7:**
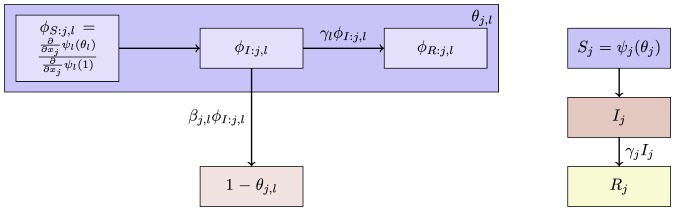
Assortative mixing by type model. We separate nodes by type. We assume that type may influence infectiousness and susceptibility as well as connections. For simplicity, we assume a finite number of groups. The resulting system is similar to our system for correlated infectiousness and susceptibility.

Figure 7 gives
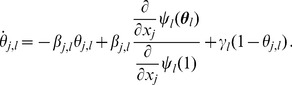
(10)The denominator 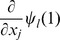 is simply the average of 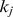 for nodes of group 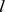. We find
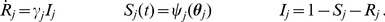
(11)


As a special case, we can consider a population where the number of partnerships a node has with one group is assigned independently of the number that node has with any other group. We set 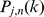 to be the probability a node of group 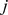 has 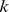 edges to a node of group 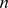 and define 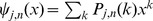. Then 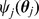 factors and may be written as 
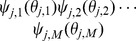
. In this special case we get
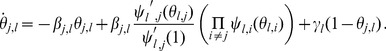
(12)


**Figure 8:**
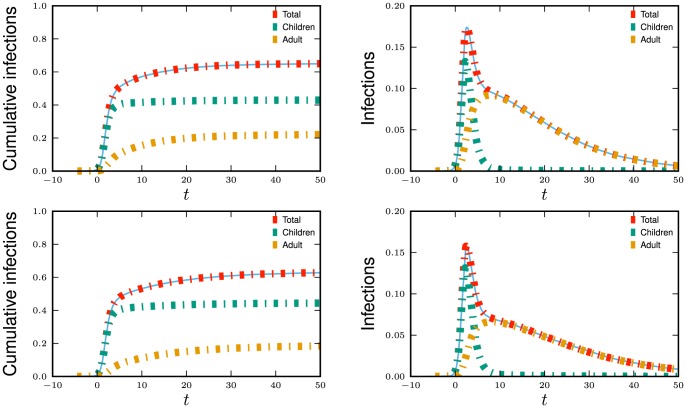
Assortative mixing by type example. Comparison of theory and simulated results for mixing with demographic groups described in section 2.2.3. We also show the predicted levels of infection in each subgroup. Simulations in a population of 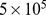 individuals (solid) and theory (dashed) are in good agreement. The difference between the top and bottom result from changing the correlations of within and between-group mixing. We choose 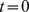 to correspond to 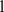
% cumulative incidence.

We now demonstrate the equations in a concrete example. To demonstrate the ability to capture demographic information, we consider a population made up of two groups, which we arbitrarily label *children* and *adults*. The between-group degrees are binomially distributed with 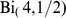, so that the average between-group degree is 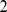. An adult's within-group degree equals its between-group degree. In contrast, a child's within-group degree is given by 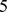 times its between-group degree. Thus people with higher between-group degree have higher within-group degree. We get
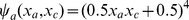


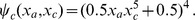
We set the disease parameters to be
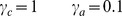


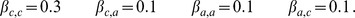
The results are shown in the top of figure 8.

We repeat with the same parameters, but this time with the correlations in degree switched so that higher between-group degrees implies lower within-group degrees. An adult's within-group degree is 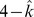 where 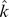 is its between-group degree. A child's within-group degree is 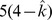 where 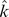 is its between-group degree. We have
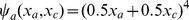


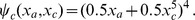
The results are shown in the bottom of figure 8.

The distribution of within and between-group partnershipss in the two populations are the same. The only distinction is that the correlations of within and between-group partnerships are different. This results in differences in the course of the epidemics. A mass action model could not distinguish between the populations.

In both cases the disease spreads quickly through the child population. Early on the spread in adults is driven largely by the explosive growth in children. Because of the correlations of adults' within and between-group degrees, those adults who are infected by children in the first scenario tend to have more adult partners and infect high-degree adults who in turn infect more high-degree children. In the second scenario however, infected children tend to infect fewer adults who tend to have fewer adult partners. The disease does not grow as quickly, but it also decays less quickly because more high degree nodes remain.

#### 2.2.4 Multiple modes of transmission

**Figure 9:**
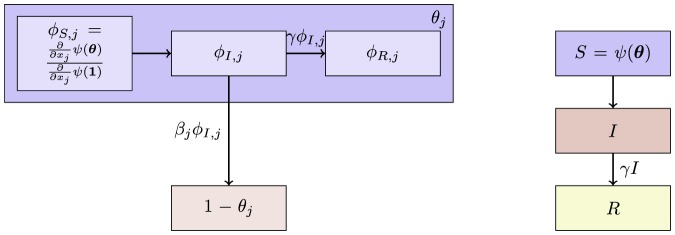
Multiple modes of transmission model. Flow diagram showing the flux of edges for the 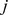-th contact type for a disease which has multiple modes of transmission.

Rather than having different types of nodes, there may be multiple modes of transmission with different mixing and infection rates for each mode. For example, HIV may spread through sexual contact and needle-sharing. The sexual contact network may have little to no relation to the needle-sharing network. Previous attempts to analyze such effects in a network context have typically assumed that there are two types of contacts: mass action-like contacts and partnerships within a network [37]. The model we derive here allows each mode of transmission to have its own underlying network structure and the equations are simpler. Assume there are 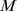 types of partnerships and that the rate of transmission along partnerships of type 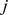 is given by 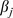. Let the joint distribution of the number of each partnership type be given by 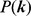 where 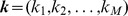. Assume recovery rates are independent of how infection was acquired. We set 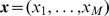 and denote the product 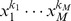 by 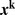. We define
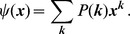
We can apply the same method to each edge type as shown in figure 9. We set 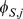 to be the probability that an edge of type 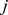 connects the test node to a susceptible partner. If 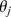 is the probability a partnership of type 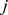 has not yet transmitted infection, we set 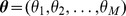. We find 
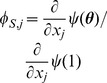
 and similarly the probability an edge of type 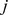 connects to a recovered partner which did not transmit is 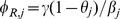. As before we find 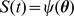. We have
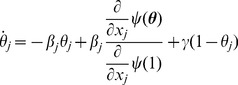
(13)

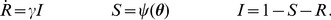
(14)In the case where the degree with respect to one partnership type is independent of that with respect to another, we can simplify equation (13) to be similar to equation (12).

**Figure 10:**
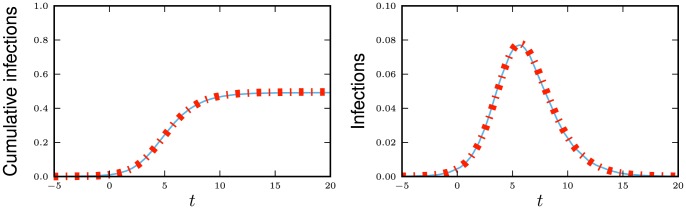
Multiple modes of transmission example. Disease spread in a population with three different types of partnerships, each with a different degree distribution described in 2.2.4. Simulations in a population of 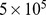 individuals (solid) and theory (dashed) are in good agreement. We choose 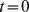 to correspond to 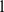
% cumulative incidence.

We now demonstrate the equations in a concrete example. We consider a population with three different types of partnerships. We take 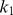, 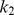, and 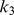 to denote the number of each type of partnership a node has. We assume that partnership type 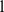 is binomially distributed with 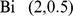 (giving a mean of 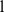). Partnership type 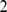 is geometrically distributed, with mean 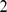. Partnership type 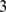 has a negative binomial distribution 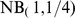 with mean 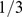 and variance 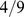. The numbers of partners an individual has of each type are assigned independently. We find
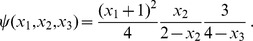
We take 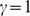 and set 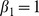, 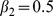, and 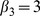 for each partnership type. We compare simulation and theory in figure 10. This example is chosen so that the variables of 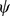 can be separated into different terms. The theory still applies even when this factorization of 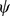 is not possible.

#### 2.2.5 Multiple infectious stages

**Figure 11:**
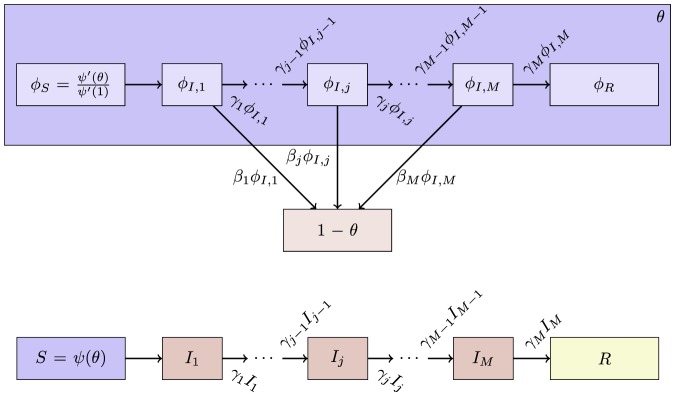
Multiple infectious stages model. Flow diagrams for a disease with several infected stages. When a disease progresses through several states (or has an infectious period that is not exponentially distributed) it is convenient to use a stage-progression model to represent the state of an edge.

There are a number of diseases with multiple infectious stages such as Tuberculosis and HIV. Some diseases begin with a non-infectious latent phase, some begin with a highly infectious acute stage before settling into a long-term chronic stage, and others oscillate between phases of high and low infectiousness. To model such situations we adapt a standard chain progression model, for which there are 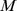 infectious phases shown in figure 11. We are not able to explicitly solve for all variables in terms of 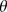, so we must find the fluxes between the compartments. We can still find 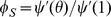, so we are able to find 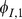 in terms of the other variables using 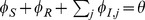. We obtain
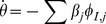
(15)

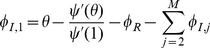
(16)

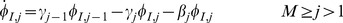
(17)

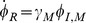
(18)

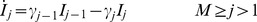
(19)

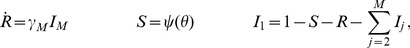
(20)where 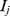 is the proportion of the population in the 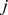-th infectious class.

**Figure 12:**
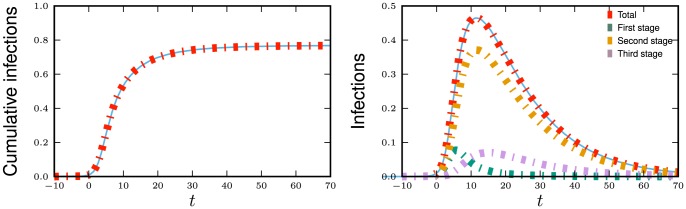
Multiple infectious stages example. The spread of the disease described in 2.2.5 with three infectious stages. Simulations in a population of 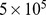 individuals (solid) and theory (dashed) are in good agreement. We choose 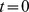 to correspond to 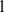
% cumulative incidence.

We now demonstrate the equations in a concrete example. Consider now the spread of a disease for which there are three infectious stages. The first stage is moderately infectious but not long, with 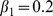, 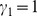. The second stage is much longer, but has a substantially lower infectiousness, with 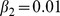 and 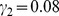. The final stage has an intermediate duration but substantially higher infectiousness, with 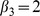 and 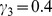. We assume that the disease spreads in a population with degree distribution 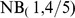 having mean 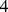 and variance 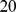 with
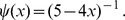
The results are shown in figure 12.

### 2.3 Dynamic Networks and Serosorting

For some diseases, it is not uncommon for individuals to actively seek out partners of similar disease status (as in HIV [38] or Leprosy [39]) or even discordant status (as in “chicken pox parties” or “swine flu parties”). This is commonly known as serosorting. To study these populations, we must use dynamic network models, which we developed in [1].

We study serosorting in two models. In the first, we use an “actual degree” model where an individual has a given number of potential partnerships, of which only a proportion are active at any given time. In the second, we use an “expected degree” model in which individuals break any existing partnerships at a fixed rate, but different individuals may find new partners at differing rates leading to a variation in the expected number of partnerships across the population.

For simplicity, we will assume that there is no recovered class and once infected an individual remains infected. This restriction is easily removed, but by using it, we are able to simplify the model and reduce the number of parameters needed. We again consider a test node, and as before we assume that if infected the test node does not cause any infections. We make an additional assumption that if infected the test node continues to behave as if it were susceptible, and that its potential partners treat it as if it were susceptible. We can think of the test node as an individual who is immune, but is unaware of that immunity, and we track the probability that the test node has not yet received a sufficient dose to infect a non-immune individual.

#### 2.3.1 Actual Degree Serosorting model

In the actual degree formulation, we think of an individual as having 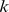 *stubs* or half-edges. These stubs may be active (and connected to another node's stub) or dormant (and available to form new edges).

When an edge breaks, the corresponding stubs enter a dormant phase. We assume that the rate a dormant edge belonging to a susceptible individual finds a new susceptible partner is 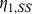, and the rate it finds a new infected partner is 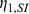. These may depend on the density of susceptible and infected individuals in the population. Similarly, active edges break at rates depending on the status of the nodes. Edges between susceptible nodes break at rate 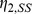, edges between a susceptible and infected node breaks at rate 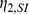, and edges between infected nodes break at rate 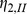.

**Figure 13:**
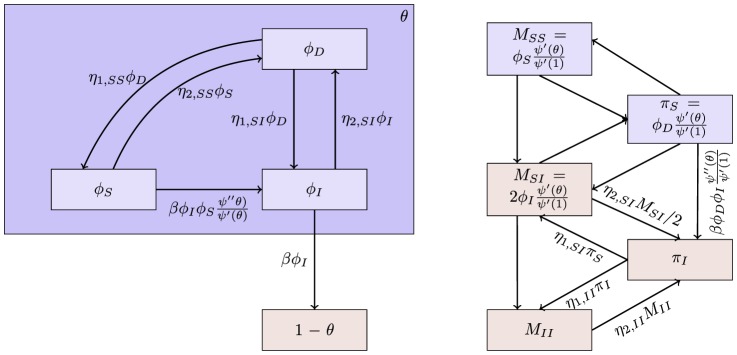
Fixed-degree serosorting model. Flow diagram showing the interplay involved in serosorting with fixed-degree. We do not consider a recovered class, which simplifies the equations. The framework can be adapted to include a recovered class. The 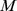 variables represent the total proportion of stubs involved in edges between the two types and the 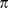 variables are the proportion of dormant stubs belonging to nodes of each type. The 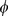 variables are as before. For the right hand side, we are able to determine most of the variables analytically, so we only need the fluxes into and out of 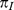. We expect that the edge breaking and rejoining rates 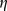 will depend on values of 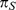 and 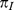.

We define 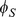, 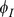, 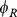, and 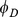 to be the probability that a stub belonging to the test node 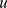 has never been part of an edge that transmitted infection to 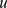, and that the stub is currently connected to a susceptible, infected, or recovered node or is dormant respectively. The fluxes between these states are shown in figure 13. Unlike in previous cases, we are unable to explicitly calculate 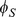, so we must track the flux into and out of 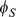. The fluxes between 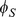 and 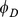 are straightforward. However, the flux from 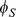 to 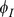 requires more attention. We repeat our derivation from [1]. Consider a partner 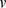 of the test node 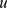 having the following properties: the stub belonging to 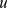 never transmitted to 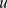 prior to joining with the stub belonging to 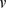, and similarly the stub belonging to 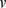 never transmitted to 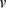 prior to joining with the stub belonging to 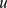. Given this, the probability 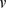 is susceptible is 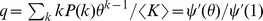. Thus, given that 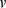 is susceptible, the rate 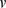 becomes infected is
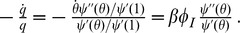
Thus the flux from 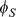 to 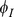 is 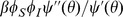, the product of 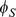, the probability that a stub has not transmitted infection to the test node and connects to a susceptible node, with 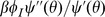, the rate that the partner becomes infected given that the stub has not transmitted and connects to a susceptible node. We need to account for the number of stubs that are in edges between different classes of nodes or are dormant. We use 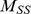 to be the proportion of all stubs that are in edges between susceptible nodes. Equivalently this is the probability that a stub is active, connects to a susceptible node, and belongs to a susceptible node: 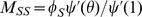. We similarly define 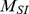 to be the proportion of all stubs that are in edges between a susceptible and infected node. We calculate this by finding the probability a stub is active, connects to an infected node, but belongs to a susceptible node but we must multiply by 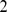 because this only counts one stub in each edge. We get 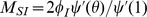 . We also define 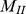 to be the proportion of all stubs in edges between infected nodes. We will calculate its value later. We set 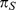 to be the proportion of stubs that are dormant and belong to susceptible nodes and 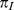 to be the proportion of stubs that are dormant and belong to infected nodes. The value of 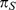 can be calculated as for the dormant contact case of [1] to be 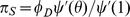. The value of 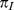 is calculated by finding the fluxes out of the other states. Once we have all of these variables, we have 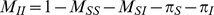.

Following figure 13 we find
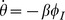
(21)

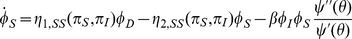
(22)

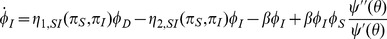
(23)

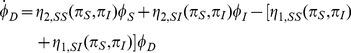
(24)

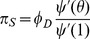
(25)

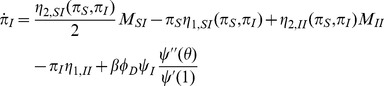
(26)

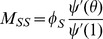
(27)

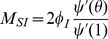
(28)

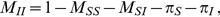
(29)where 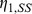, 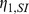, 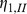, 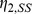, 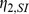, and 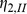 are likely to depend on 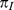 and 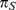 and may depend on the other quantities. What form that dependence takes is determined by the behavior of the population. This completes the derivation of the equations for the actual degree formulation of serosorting.

#### 2.3.2 Variable Degree Serosorting model

In many populations, it is reasonable to assume that individuals create and break contacts without regard to whether contacts already exist. Consequently, the concept of having a fixed number of stubs is inappropriate. For these populations, we assume that in the absence of disease all contacts will have the same expected duration but different individuals will create new contacts at different rates, resulting in some having higher or lower average degrees. In [1], we used 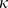 to be the *expected degree* of a node. However, when behavior changes based on infection status, the expected degree of individuals can change. Instead, we refer to 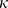 as the *desired* degree because depending on how sorting happens, it may not be possible for a node to have expected degree 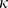. However, 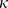 will represent the expected degree of an individual if there were no infection present. We again use 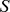 and 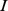 to be proportions of the population. 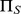 and 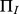 measure the proportion of desired contacts which belong to susceptible or infected individuals: 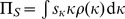, 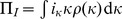 where 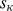 and 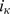 denote the proportion of individuals with desired degree 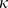 who are susceptible and infected respectively.

We assume that the population behavior proceeds as before, but an uninfected node will end and form contacts with different rates for infected or susceptible partners. There are many ways in which this could be modeled. We will assume that a susceptible individual with desired degree 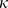 acquires new susceptible contacts at rate 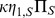 and new infected contacts at rate 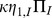, where both 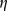 parameters may depend on 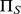 and 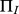. Similarly, a susceptible individual will end an existing contact with another susceptible and with an infected individual at rates 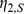 and 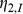 respectively (where again both 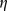 parameters may depend on 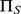 and 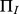). We assume that 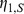 and 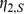 are equal if 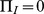 so that in a disease-free population an individual's expected and desired degrees coincide.

We need to add variables in order to track the probability of having existing edges connecting to susceptible or infected nodes. Consider a test node 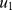 with desired degree 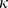, and another 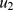 with desired degree 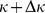. We define 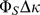 to be the expected additional number of edges to susceptible partners that 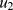 would have and 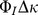 to be the expected additional number of edges to infected partners which have not transmitted that 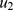 would have. We take the values of 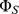 and 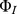 in the 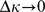 limit. In the cases considered in [1], the value of 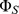 and 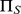 were the same. However, because there is active selection of partner based on disease status, in this case 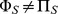.

**Figure 14:**
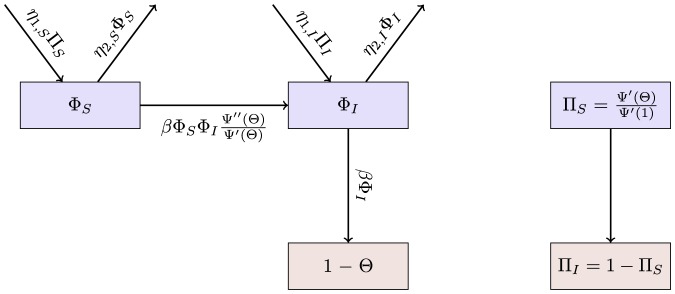
Variable-degree serosorting model. Flow diagram showing the interplay involved in serosorting. We do not consider a recovered class, which simplifies the equations significantly. The framework can be adapted to include a recovered class. The 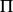 variables give the proportion of contacts that would be formed with susceptible or infected individuals assuming that their behavior is not altered by disease. The 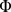 variables are the probability that a current contact of the test node is with an individual of given type, under the assumption that the test node always behaves as if susceptible, and does not transmit to its partners. We expect that the edge breaking and rejoining rates 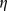 will depend on values of 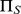 and 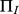. Note that 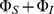 need not equal 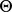.

The resulting flow diagram is shown in figure 14. We must find the flux from 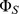 to 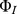. Consider a random test node 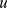 and look at a randomly chosen susceptible partner 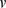. Given the desired degree 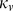 of 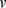, the rate that 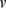 becomes infected is 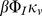. We need to determine the expected value of 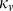 given that 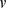 is a susceptible partner of 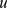. We first note that the probability density function for the partner to be susceptible and have degree 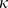 is proportional to 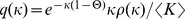 with some proportionality constant 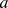. So in order to calculate the expected value of the desired degree we take 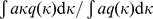. This simplifies to 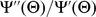. So the flux from 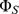 to 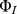 due to infection of the partner is 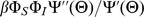


We find
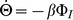
(30)

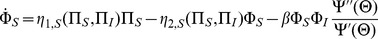
(31)

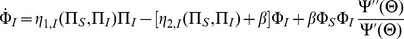
(32)

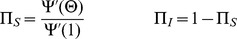
(33)

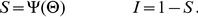
(34)So an SI epidemic in a population with serosorting can be captured by a system with just three ODEs.

## Discussion

We have applied the edge-based compartmental model approach introduced in [1] to diseases and populations with different structures, showing how to derive models for a range of scenarios. We have shown that the models derived accurately reproduces simulated epidemics in large populations with CM-like structure. With the exception of serosorting we focused our attention on static CM networks. We have considered each variation in isolation. However it is possible to adapt the approach to a disease for which several of these issues are considered simultaneously in any of the network classes discussed in [1].

In general, we can adapt most existing mean-field/mass-action style SIR models in a closed population to the spread of infectious disease through a network. When we do this, we get a 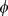 variable corresponding to each of the 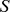, 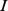, or 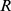 variables in the usual model. We take the usual flow diagram for 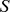, 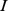, and 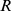 and adapt it to give the fluxes between the 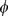 variables. We add one more compartment 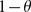, and flux goes from each of the potentially infectious 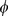 variables to 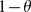. This approach produces an accurate model for disease spread through the modeled population.

## References

[1] Miller JC, Slim AC, Volz EM (2012) Edge-based compartmental modelling for infectious disease spread. Journal of the Royal Society Interface 9: 890–906. 10.1098/rsif.2011.0403 PMC3306633 21976638

[2] Miller JC (2011) A note on a paper by Erik Volz: SIR dynamics in random networks. Journal of Mathematical Biology 62: 349–358. 20309549 10.1007/s00285-010-0337-9

[3] Volz EM (2008) SIR dynamics in random networks with heterogeneous connectivity. Journal of Mathematical Biology 56: 293–310. 17668212 10.1007/s00285-007-0116-4 PMC7080148

[4] Decreusefond L, Dhersin JS, Moyal P, Tran VC (2012) Large graph limit for an SIR process in random network with heterogeneous connectivity. The Annals of Applied Probability 22: 541–575.

[5] Anderson RM, May RM (1991) Infectious Diseases of Humans. Oxford: Oxford University Press.

[6] May RM, Anderson RM (1988) The transmission dynamics of human immunodeficiency virus (HIV). Phil Trans R Soc Lond B 321: 565–607. 2907158 10.1098/rstb.1988.0108

[7] May RM, Lloyd AL (2001) Infection dynamics on scale-free networks. Physical Review E 64: 066112. 10.1103/PhysRevE.64.066112 11736241

[8] Moreno Y, Pastor-Satorras R, Vespignani A (2002) Epidemic outbreaks in complex heterogeneous etworks. The European Physical Journal B-Condensed Matter and Complex Systems 26: 521–529.

[9] Pastor-Satorras R, Vespignani A (2001) Epidemic spreading in scale-free networks. Physical Review Letters 86: 3200–3203. 11290142 10.1103/PhysRevLett.86.3200

[10] Eames K, Keeling M (2002) Modeling dynamic and network heterogeneities in the spread of sexually transmitted diseases. Proceedings of the National Academy of Sciences 99: 13330–13335. 10.1073/pnas.202244299 PMC130633 12271127

[11] Eames K, Keeling M (2006) Coexistence and specialization of pathogen strains on contact networks. The American Naturalist 168: 230–241. 10.1086/505760 16874632

[12] Bauch C (2002) A versatile ode approximation to a network model for the spread of sexually transmitted diseases. Journal of mathematical biology 45: 375–395. 12424529 10.1007/s002850200153

[13] House T, Davies G, Danon L, Keeling MJ (2009) A motif-based approach to network epidemics. Bulletin of Mathematical Biology 71: 1693–1706. 19396497 10.1007/s11538-009-9420-z

[14] House T, Keeling M (2011) Insights from unifying modern approximations to infections on networks. Journal of The Royal Society Interface 8: 67–73. 10.1098/rsif.2010.0179 PMC3024819 20538755

[15] Miller JC, Kiss IZ (2013) Epidemic spread in networks: Existing methods and current challenges. Under Review 10.1051/mmnp/20149202 PMC4287241 25580063

[16] Ball F, Neal P (2008) Network epidemic models with two levels of mixing. Mathematical Biosciences 212: 69–87. 18280521 10.1016/j.mbs.2008.01.001

[17] Lindquist J, Ma J, van den Driessche P, Willeboordse F (2011) Effective degree network disease models. Journal of Mathematical Biology 62: 143–164. 20179932 10.1007/s00285-010-0331-2

[18] Diekmann O, De Jong MCM, Metz JAJ (1998) A deterministic epidemic model taking account of repeated contacts between the same individuals. Journal of Applied Probability 35: 448–462.

[19] Altmann M (1998) The deterministic limit of infectious disease models with dynamic partners. Mathematical Biosciences 150: 153–175. 9656648 10.1016/s0025-5564(98)00012-1

[20] Dietz K, Tudor D (1992) Triangles in heterosexual hiv transmission. AIDS epidemiology: methodological issues 143–155.

[21] Dietz K, Hadeler K (1988) Epidemiological models for sexually transmitted diseases. Journal of Mathematical Biology 26: 1–25. 3351391 10.1007/BF00280169

[22] Lloyd-Smith JO, Getz WM, Westerhoff HV (2004) Frequency-dependent incidence in models of sexually transmitted diseases: portrayal of pair-based transmission and effects of illness on contact behaviour. Proceedings of the Royal Society of London Series B: Biological Sciences 271: 625–634. 15156921 10.1098/rspb.2003.2632 PMC1691637

[23] Wallinga J, Teunis P, Kretzschmar M (2006) Using data on social contacts to estimate age-specific transmission parameters for respiratory-spread infectious agents. American Journal of Epidemiology 164: 936. 16968863 10.1093/aje/kwj317

[24] Volz E, Frost S, Rothenberg R, Meyers L (2010) Epidemiological bridging by injection drug use drives an early HIV epidemic. Epidemics 2: 155–164. 21352786 10.1016/j.epidem.2010.06.003

[25] Pilcher C, Tien H, Eron J, Vernazza P, Leu S, et al (2004) Brief but efficient: acute HIV infection and the sexual transmission of HIV. Journal of Infectious Diseases 189: 1785. 15122514 10.1086/386333

[26] Miller JC Epidemics on networks with large initial conditions or changing structure. Under Review 10.1371/journal.pone.0101421 PMC4086930 25004149

[27] Molloy M, Reed B (1995) A critical point for random graphs with a given degree sequence. Random Structures & Algorithms 6: 161–179.

[28] Newman MEJ (2003) The structure and function of complex networks. SIAM Review 45: 167–256.

[29] Newman MEJ, Strogatz SH, Watts DJ (2001) Random graphs with arbitrary degree distributions and their applications. Physical Review E 64: 026118. 10.1103/PhysRevE.64.026118 11497662

[30] van der Hofstad R (2010) Random Graphs and Complex Networks. URL http://www.win.tue.nl/~rhofstad/NotesRGCN.pdf. Author's website: accessed 18 Jun 2013.

[31] Miller JC, Volz EM (2012) Model hierarchies in edge-based compartmental modeling for infectious disease spread. Journal of Mathematical Biology 1–31. 10.1007/s00285-012-0572-3 PMC3552133 22911242

[32] Meyers LA, Newman M, Pourbohloul B (2006) Predicting epidemics on directed contact networks. Journal of Theoretical Biology 240: 400–418. 16300796 10.1016/j.jtbi.2005.10.004

[33] Sharkey K, Fernandez C, Morgan K, Peeler E, Thrush M, et al (2006) Pair-level approximations to the spatio-temporal dynamics of epidemics on asymmetric contact networks. Journal of Mathematical Biology 53: 61–85. 16791650 10.1007/s00285-006-0377-3

[34] Miller JC (2007) Epidemic size and probability in populations with heterogeneous infectivity and susceptibility. Physical Review E 76: 010101(R). 10.1103/PhysRevE.76.010101 17677396

[35] Ball F, Clancy D (1995) The final outcome of an epidemic model with several different types of infective in a large population. Journal of Applied Probability 579–590.

[36] Ball F (1985) Deterministic and stochastic epidemics with several kinds of susceptibles. Advances in Applied Probability 17: 1–22.

[37] Ball F, Neal P (2002) A general model for stochastic SIR epidemics with two levels of mixing. Mathematical Biosciences 180: 73–102. 12387917 10.1016/s0025-5564(02)00125-6

[38] Parsons J, Schrimshaw E, Wolitski R, Halkitis P, Purcell D, et al (2005) Sexual harm reduc- tion practices of HIV-seropositive gay and bisexual men: serosorting, strategic positioning, and withdrawal before ejaculation. AIDS 19: S13. 10.1097/01.aids.0000167348.15750.9a 15838191

[39] Leviticus, chapter 13.

